# Combining Soft Polysilazanes with Melt-Shear Organization of Core–Shell Particles: On the Road to Polymer-Templated Porous Ceramics

**DOI:** 10.3390/molecules24193553

**Published:** 2019-09-30

**Authors:** Anna K. Boehm, Emanuel Ionescu, Marcus Koch, Markus Gallei

**Affiliations:** 1 Chair in Polymer Chemistry, Saarland University, Campus Saarbrücken C4 2, 66123 Saarbrücken, Germany; annakatharina.boehm@uni-saarland.de; 2 Department of Materials and Earth Sciences, Technische Universität Darmstadt, Otto-Berndt-Str. 3, 64287 Darmstadt, Germany; ionescu@materials.tu-darmstadt.de; 3 INM- Leibniz Institute for New Materials, Campus D2 2, Saarland University, 66123 Saarbrücken, Germany; marcus.koch@leibniz-inm.de

**Keywords:** polymer particle synthesis, particle processing, polymer chemistry, preceramic materials, melt-shear organization, polymer derived ceramic, core–shell

## Abstract

The preparation of ordered macroporous SiCN ceramics has attracted significant interest and is an attractive area for various applications, e.g., in the fields of catalysis, gas adsorption, or membranes. Non-oxidic ceramics, such as SiCN, own a great stability based on the covalent bonds between the containing elements, which leads to interesting properties concerning resistance and stability at high temperature. Their peculiar properties have become more and more important for a manifold of applications, like catalysis or separation processes, at high temperatures. Within this work, a feasible approach for the preparation of ordered porous materials by taking advantage of polymer-derived ceramics is presented. To gain access to free-standing films consisting of porous ceramic materials, the combination of monodisperse organic polymer-based colloids with diameters of 130 nm and 180 nm featuring a processable preceramic polymer is essential. For this purpose, the tailored design of hybrid organic/inorganic particles featuring anchoring sites for a preceramic polymer in the soft shell material is developed. Moreover, polymer-based core particles are used as sacrificial template for the generation of pores, while the preceramic shell polymer can be converted to the ceramic matrix after thermal treatment. Two different routes for the polymer particles, which can be obtained by emulsion polymerization, are followed for covalently linking the preceramic polysilazane Durazane1800 (Merck, Germany): (i) Free radical polymerization and (ii) atom transfer radical polymerization (ATRP) conditions. These hybrid hard core/soft shell particles can be processed via the so-called melt-shear organization for the one-step preparation of free-standing particle films. A major advantage of this technique is the absence of any solvent or dispersion medium, enabling the core particles to merge into ordered particle stacks based on the soft preceramic shell. Subsequent ceramization of the colloidal crystal films leads to core particle degradation and transformation into porous ceramics with ceramic yields of 18–54%.

## 1. Introduction

Polymer-derived ceramics (PDCs) and nanocomposites emerged in the last decades as high-potential materials with unique phase compositions and microstructures, as well as outstanding structural and functional properties [1,2,3,4,5,6,7,8]. Their preparative access from liquid or soluble preceramic polymers allows the use of various processing/shaping techniques to prepare (nano)powders, fibers, coatings, or monolithic parts. Among those, PDCs with designed micro/meso/macro porosity were developed for various purposes, such as gas separation, catalyst supports, thermal insulation, drug delivery, etc. [9,10,11,12,13,14,15,16,17]. Especially, macroporous non-oxidic polymer-derived ceramics such as SiC, Si_3_N_4_, and SiCN were developed as robust, high-temperature capable materials [18,19,20,21,22,23,24].

Various processing techniques have been used to achieve macroporosity in ceramic materials in general, and in PDCs in particular, e.g., direct foaming, replica methods, as well as the use of porogens (sacrificial fillers) [13,25,26]. As for specific applications, the presence of ordered porosity is required, and several approaches have been used to prepare ordered macroporous PDCs, such as lithography [27], additive manufacturing [28,29,30,31], or freeze casting [16,32,33]. Recently, the use of sacrificial fillers to generate ordered porosity in PDCs was proposed. Typically, particulate sacrificial polymeric fillers such as polystyrene (PS) [22,34,35], polymethylmethacrylate (PMMA) [36,37,38], polyethylene (PE) [39,40], or polyvinyl alcohol (PVA) [41] have been used. Ordered porosity may be achieved by (i) providing a stable colloidal mixture consisting of preceramic polymer and fillers, (ii) slow evaporation of the solvent, which occurs accompanied by a close(st) packed assemblage of the filler particles, and subsequently (iii) thermal treatment leading to ceramization of the preceramic polymer and the burn-out of the filler particles [2,35,42,43].

An alternative preparation of ordered macroporous PDCs considers the melt-shear organization of soft-shell/hard-core hybrid material formulations [44,45,46,47,48,49]. Within this context, the hard core can consist of a sacrificial material (such as PS, PMMA, etc.), which is removed during the subsequent thermal treatment, whereas the soft shell consists of a preceramic polymer, which is converted into ceramic during the thermal treatment. Compared to other preparation methods, e.g., electrosprayed core–shell nanoparticles, emulsion polymerization provides access to complex particle architectures in one step based on the phenomenon of incompatibility between different polymers [50,51,52,53]. The challenge within this context relates to the covalent binding of the preceramic polymer onto the particle surface and the cross-linking and ceramization behavior of the preceramic polymers, which has to be carefully controlled. For the purpose of the melt-shear assembly of the core particles, the preceramic polymer should be soft at the beginning of the process, in order to allow their closest-packed arrangement by core/shell particle merging. However, the polymer should be cured prior to ceramization in order to make it infusible and thus able to maintain the shape during the polymer-to-ceramic conversion.

As for polysilazanes, their cross-linking and hardening behavior has been intensively studied in the past [54]. It was shown that the cross-linking of polysilazanes may occur via dehydrocoupling processes (Si–H/Si–H or Si–H/N–H) or, in the case that vinyl (or alkenyl) and Si–H groups are present, via hydrosilylation. Additionally, vinyl (alkenyl) polymerization can be used to cure the polysilazanes [54]. All mentioned methods to cross-link polysilazanes cannot be properly controlled, thus, once the process starts, it occurs until completion, resulting in an infusible, highly cross-linked polysilazane. This is rather disadvantageous when, e.g., the preparation of ceramic fibers is anticipated, which requires that the preceramic polymer is still meltable after the cross-linking step. This was recently achieved by using a tetra-*n*-butylammonium-fluoride-catalyzed Si–H/N–H dehydrocoupling process, which can be controlled very well upon termination with Ca(BH_4_)_2_ [55,56].

Similarly, the melt-shear organization to be used in the present study requires a covalent bond of the preceramic polymer on the surface of the polymer-based cores and a strict control of the cross-linking of the polysilazane. In the present work, vinyl polymerization was used to bind the polysilazane to the surface of monodisperse polymer-based core particles prepared via emulsion polymerization. For further processing of the hybrid core–shell particles through melt-shear organization, it is important to have a meltable, i.e., soft polysilazane shell [45]. Due to these basic prerequisites, the vinyl polymerization should take place in a defined way to prevent a too extensive cross-linking of the polysilazane, which comes with issues during processing. On the other hand, too few cross-linking sites of the soft shell would lead to a detachment of the shell material during extrusion and processing via melt-shearing [57,58]. In more detail, the second challenge to be considered in the present study relates to the covalent bonding of the polysilazane to the core particles.

Concerning the polymer-based core particles, many monomers are suitable for starved-feed emulsion polymerization leading to monodisperse particles. In our work, we chose colloids mainly based on poly(methyl methacrylate) (MMA) as, in general, these materials feature good degradation behavior with respect to full removal of the sacrificial core materials.

**Figure 1:**
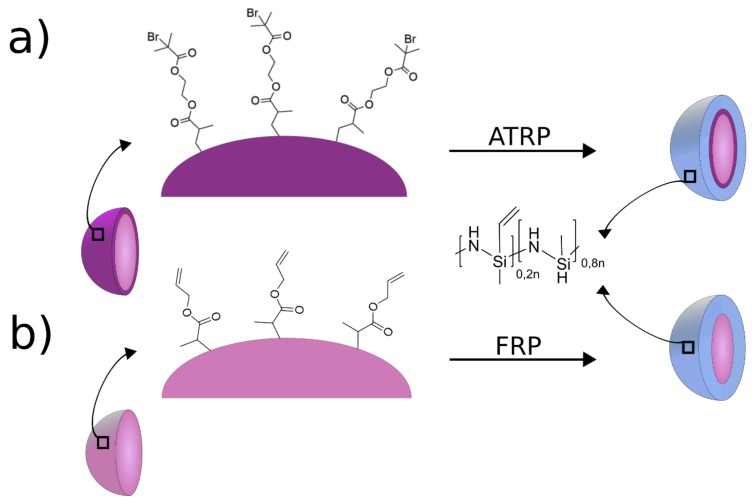
Functional groups on the particle surface of synthesized monodisperse colloids for the binding of polysilazane via atom transfer radical polymerization (ATRP) (**a**) and free radical polymerization (FRP) (**b**).

Thus, the first strategy to prepare hybrid core–shell particles was based on free radical polymerization (FRP). Therefore, particles with free C=C double bounds were considered for this step. However, as FPR may lead also to an extensive cross-linking of the polysilazane itself (which is not desired at this stage), a grafting-from strategy was further chosen for building a meltable polysilazane shell around the polymer-based core particles. With an atom transfer radical polymerization (ATRP) initiator on the surface of the particles, it is possible to create a weakly cross-linked polysilazane shell at the particle surface, while extensive cross-linking of the polysilazane itself (like in the case of FRP) is suppressed. The covalent binding between functional groups of a particle surface and a polysilazane has not been reported for the preparation of structured and porous SiCN ceramic materials. Within the present study, we report, for the first time, the covalent binding of the preceramic polysilazane Durazane1800 to the surface of organic template particles, which are accessible via emulsion polymerization. Subsequent surface functionalization protocols comprising FRP and atom transfer radical polymerization (ATRP) for immobilization of the polysilazane shell are depicted in Figure 1. The linkage of the soft shell to the comparably hard core is investigated in more detail, as it is one of the basic prerequisites for the melt-shear organization technique.

For both methods, organic particle cores with defined functional groups for each strategy were prepared via starved-feed emulsion polymerization. This technique allows the synthesis of seed shell architectures, while the shell contains the functional groups for the covalent binding of the polysilazane. Polysilazane immobilization and application of the melt-shear organization technique yields free-standing preceramic opal films, while ceramization leads to ceramic inverse films with ordered open-porous structures.

## 2. Results and Discussion

**Figure 2:**
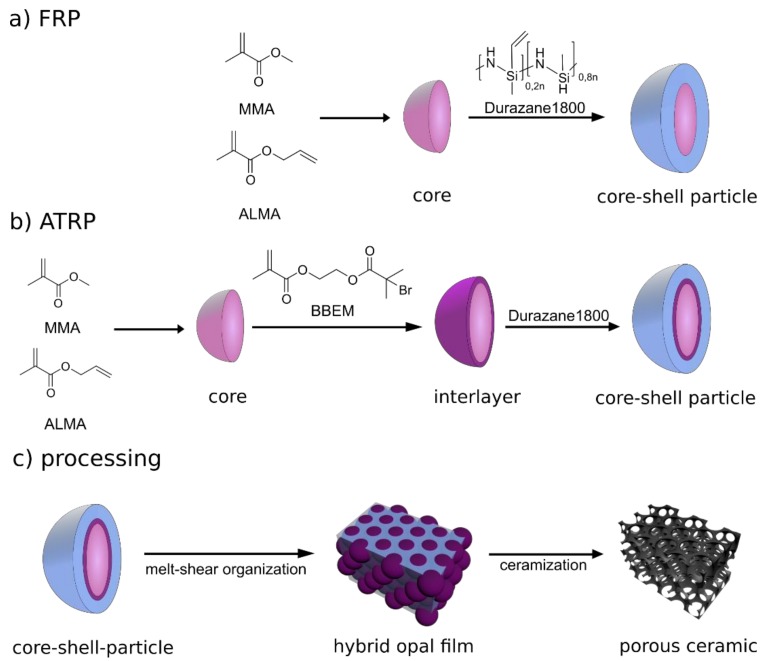
Hybrid organic/inorganic preceramic core–shell particle synthesis via emulsion polymerization and FRP (**a**)/ATRP (**b**) and processing (**c**) into hybrid opal films through melt-shear organization, followed by thermal conversion into highly ordered porous SiCN ceramic.

In the present work, the preparation of ordered porous SiCN-based ceramic structures was realized upon using a preceramic polysilazane as a precursor and the melt-shear organization of colloidal soft-shell/hard-core hybrid particles (Figure 2).

Monodisperse polymeric particles prepared through the starved-feed emulsion polymerization were used as templates for well-defined pores. The use of simple physical blends consisting of the polymer particles and the commercially available polysilazane did not lead to hybrid opal film morphologies during melt-shearing, which were required to generate the ordered porous ceramic structures. Through this process, there was no homogenous embedding of the particles into the polysilazane as a matrix, emphasizing that a more defined immobilization of the soft shell to the organic core particles is necessary. In detail, for the blend system, the organic particles were observed in the middle of the film, while the viscous polysilazane flowed to the outside of the film. Furthermore, the film was too soft to separate it after application of the melt-shear process and no free-standing material could be obtained.

For that reason, it was necessary to fabricate hybrid core–shell particles with immobilization sites for polymer core and a polysilazane shell in order to impede a core–shell separation during processing. As Durazane1800 is inappropriate for the emulsion polymerization, the combination of the emulsion polymerization with other polymerization techniques was necessary, and grafting strategies were taken into account. According to this principle, the creation of functional, monodisperse particles, capable for further reactions, such as free radical polymerization (FRP) or ATRP, is essential. This leads to hybrid colloids containing an organic, polymer-based core and an inorganic shell made of a ceramic precursor. These hybrid core–shell particles were suitable to form hybrid opal films using the melt-shear organization due to the monodisperse and cross-linked polymer-based colloid core and the covalently bound and meltable preceramic shell. These circumstances led to pressure-induced self-assembled core-particle stacks, as reported previously for the preparation of elastomeric opal films [43,49,59,60]. Hybrid opal films could be subsequently converted into porous ceramics by a ceramization process, which will be described in the ensuing sections.

As shown in Figure 2, two different types of particles were prepared for both intended polymerization methods, i.e., FRP and ATRP, to post-modify the comparably hard organic core particles with a soft polysilazane shell material. For FRP, accessible double bonds on the surface of the particles were used to bind the polysilazane. In the case of ATRP, the surface of the particles was functionalized with an ATRP inimer, leading to a grafting from linkage of the polysilazane onto the particle surface in a subsequent step.

### 2.1. Preparation of Porous SiCN -Ceramics via Free Radical Polymerization (FRP)

#### 2.1.1. Particle Synthesis via Starved-Feed Emulsion Polymerization

For the preparation of monodisperse colloids, the starved-feed emulsion polymerization technique was used. This convenient type of emulsion polymerization is a semi-continuous process, where the polymerization rate is higher than the addition of the monomer emulsion. This leads to a high control with respect to particle size, composition, and architecture [61,62,63]. In the case of FRP, cross-linked colloids made of methyl methacrylate and 30% allyl methacrylate, P(MMA-*co*-ALMA), were used. The advantage of using poly(methacrylates) lies in their good thermal degradation behavior, and has been previously used in our work [47]. The organic core particle as part of the final colloidal crystal structure was considered as the template and could be removed through a thermal degradation.

**Figure 3:**
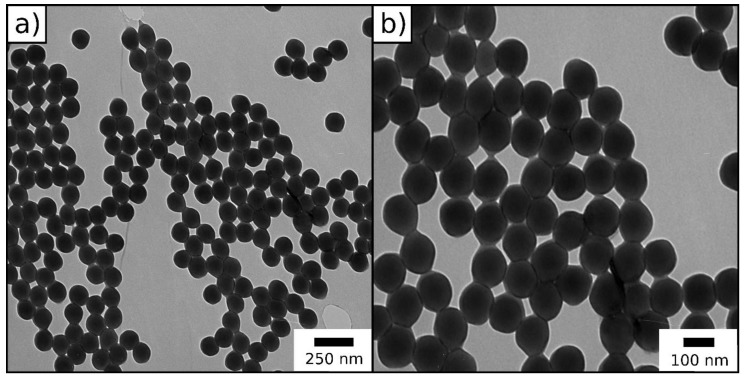
(**a**,**b**) Transmission electron microscopy (TEM) images of P(MMA-*co*-ALMA) particles.

The synthesized particles were characterized by dynamic light scattering (DLS) (Figure S1) and transmission electron microscopy (TEM) in order to demonstrate their monodispersity and assess their size (Figure 3, Table 1).

In summary, both DLS and TEM measurements give proof for the successful synthesis of monodisperse particles, which is important for further processing of the particles through melt-shearing. By comparison, DLS measurements in general give a larger diameter for particle systems in contrast to the TEM images, because the hydrodynamic diameter of the particles is determined via DLS, which turns out to be larger compared to the diameter of dried particles.

#### 2.1.2. Free Radical Polymerization of Durazane1800 on P(MMA-*co*-ALMA) Particles

**Figure 4:**
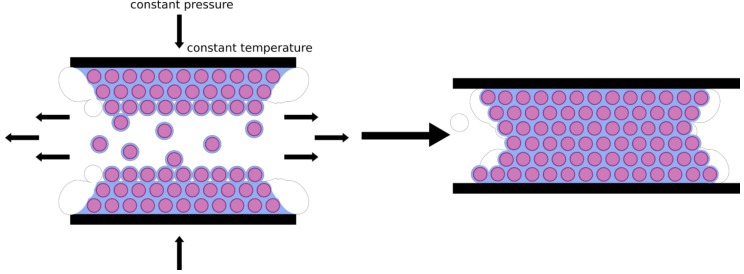
Fabrication of colloidal crystals by using the melt-shear technique.

For the free radical polymerization of the allyl groups on the colloid surface with the vinyl groups of the polysilazane, lyophilized particles were dissolved in toluene, and di-*tert*-butyl peroxide was used as an initiator, as peroxides are suitable for cross-linking of polysilazanes [64,65,66]. After free radical polymerization at 100 °C, toluene was removed under vacuum. The core–shell particles were transformed into a colloidal crystal using the melt-shear technique (Figure 4) at 160 bar and 160 °C.

**Figure 5:**
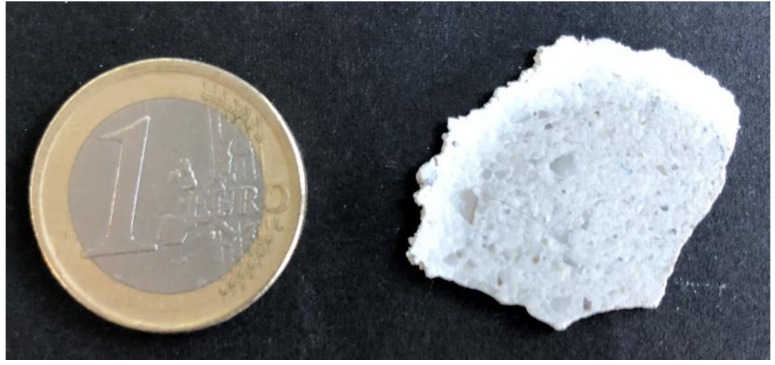
Photograph of a free-standing particle-based film processed through melt-shear organization.

While the cross-linked P(MMA-*co*-ALMA) cores keep their shape, the soft preceramic shell starts flowing and builds a matrix for the organic core particles. In comparison to other methods leading to colloidal crystals, the melt-shear organization procedure provides access to free-standing particle-based films (see exemplary photograph in Figure 5). The diameter of the obtained films is about 4 cm while ca. 1 g of hybrid core–shell particles is applied for melt-shear processing.

**Figure 6:**
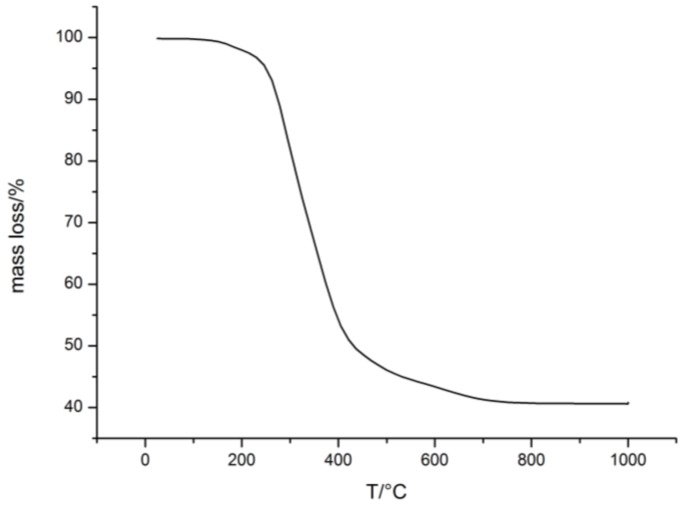
Mass loss of the colloidal crystal made of particles from FRP during the thermal treatment.

To obtain a porous ceramic from the colloidal crystal, as obtained via the free-radical polymerization strategy for polysilazane immobilization, a small amount of the film (about 15 mg) was thermally treated at 1000 °C in inert gas atmosphere (TGA measurement of the ceramization process in Figure 6).

**Figure 7:**
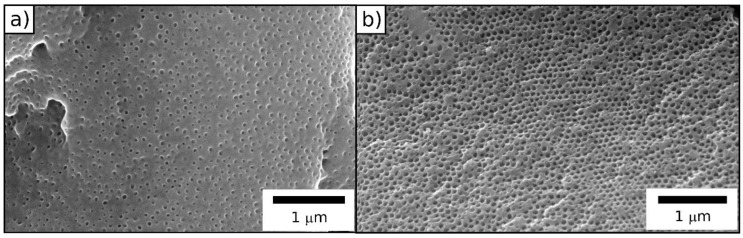
SEM photographs of the (**a**) surface and (**b**) cross-section of a porous SiCN film after melt-shearing and ceramization of core/shell particles processed by emulsion polymerization and FRP.

During this process, the polysilazane-based matrix converted into ceramic silicon carbonitride (SiCN), whereas the polymeric core particles degraded, leaving ordered pores (SEM images in Figure 7). Degradation starts at ca. 200 °C, which shows conformity with the literature [67]. The ceramic yield amounts to ca. 41%.

SEM images (Figure 7) indicate the successful preparation of an ordered porous ceramic. While the surface is partially sealed, the cross-section is completely porous, and the diameter of the pores is determined to be 60 nm. In comparison to the original particle size, the pores have smaller diameters, which shows the effect of the material shrinkage during the ceramization process.

### 2.2. Preparation of Porous SiCN Ceramics via ATRP

After successful processing of core–shell particles through free radical polymerization, ATRP as another polymerization techniques for the construction of well-defined core–shell particles containing Durazane1800 will be compared. In general, ATRP can be considered to be more defined with respect to addressable initiator moieties and, hence, for more defined linkages of polysilazane to the organic core material [68,69]. In this case, ATRP was used in order to obtain hybrid core–shell particles (Figure 1).

#### 2.2.1. ATRP Inimer Particle Synthesis via Starved-Feed Emulsion Polymerization

**Figure 8:**
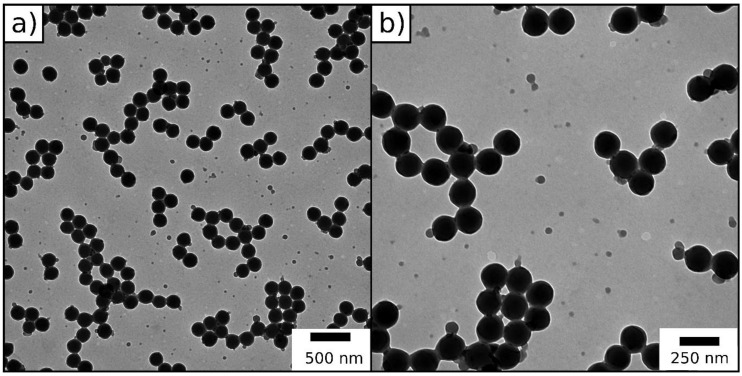
(**a**,**b**) TEM images of the inimer-containing particles.

For the preparation of monodisperse, the inimer-containing particles starved-feed emulsion polymerization technique was used. The particles consisted of a P(MMA-*co*-ALMA) core and a thin shell of the ATRP inimer 2-(2-bromoisobutyryloxy)ethyl methacrylate (BBEM). To avoid major secondary nucleation, the monomer emulsion containing the inimer was continuously added to the particle dispersion in starved-feed mode. The synthesized particles were characterized concerning their particle size distribution and size by DLS (Figure S2) and TEM (Figure 8, Table 2). We note that, besides the successful formation of spherical core/shell particles, some secondary nucleation during preparation of the inimer-shell particles occurred, which is due to the fact that the ATRP inimer cannot be fully dissolved in the added monomer/inimer emulsion.

DLS measurements give proof for the successful synthesis of well-defined ATRP inimer particles. TEM images show a few smaller particles, originated by secondary nucleation during starved-feed emulsion polymerization caused by the intrinsic properties of the inimer BBEM. Nevertheless, in the next section, these novel particles will be used for polysilazane immobilization and particle processing.

#### 2.2.2. Grafting from ATRP of Durazane1800 on the Surface of BBEM-Containing Particles

**Figure 9:**
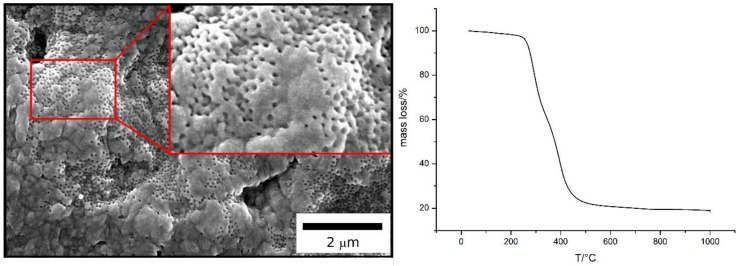
SEM photograph of the cross-section of a porous SiCN film after melt-shearing and ceramization of core/shell particles processed by emulsion polymerization and ATRP. Degradation of the polymer cores starts at ca. 200 °C, ceramic yield amounts to 18 %.

For the ATRP of Durazane1800, [Cu^I^(PMDETA)Br] was chosen as a ligand due to its high activation rate in ATRP [68,69]. This is necessary because the vinylic groups of Durazane1800 were considered to feature a rather low reactivity. As solvent, anisole was chosen since it is a common solvent for ATRP reactions. However, in order to isolate the particles for intended processing, it was necessary to precipitate them from their dispersion in anisole. The first experiments with this solvent/particle system did not lead to porous structures. Only through an excess of the polysilazane was a porous structure observed (Figure 9, TGA measurement of the ceramization process and SEM image).

As a result of the excess of the polysilazane, there are thick filled interstices between the pores. This is a result of too much polysilazane matrix material impeding the preparation of the intended open-porous structure after thermal treatment. However, as mentioned above, the addition of more polysilazane for immobilization to the organic cores was necessary for particle processing. This additionally led to a decrease of the degree of order for the particles, which was also reflected by the positions of the final pores within the thermally-treated ceramic film. As a first conclusion here, the amount of polysilazane has to be reduced, while maintaining the capability of melt-shearing. It is assumed that the core–shell ratio was inappropriate. Moreover, it seemed that anisole could not be fully removed during particle precipitation, and the residual solvent causes problems during melt-shearing. To underpin this assumption, the organic solvent was changed from anisole to THF, which features a lower boiling point. Another advantage of THF as solvent is the higher reactivity within the ATRP system because of its higher polarity [70].

**Figure 10:**
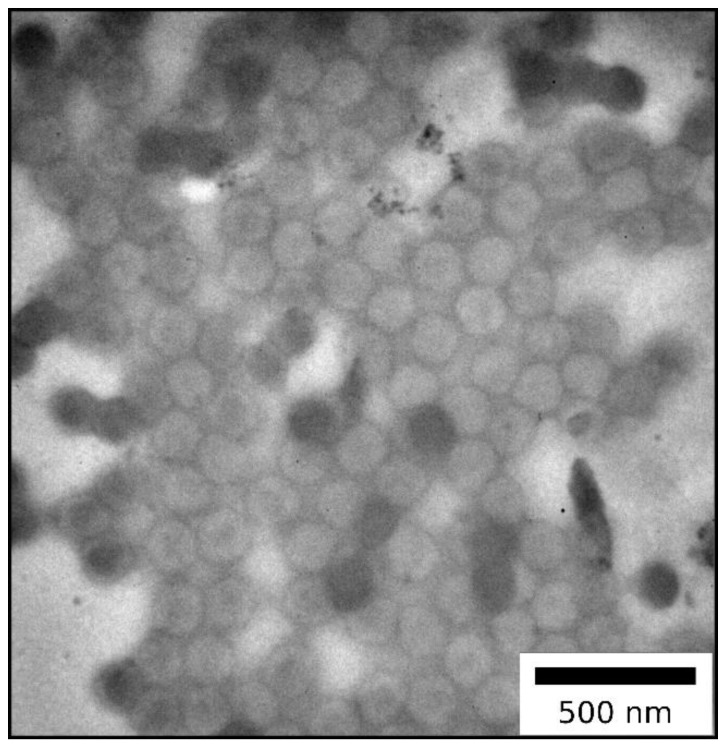
TEM image of hybrid core–shell particles consisting of inimer-containing particles featuring a Durazane1800 shell.

As described in the Experimental Section, hybrid core–shell particles featuring a polysilazane shell percentage of 60 wt.% were synthesized using ATRP (TEM image in Figure 10).

**Figure 11:**
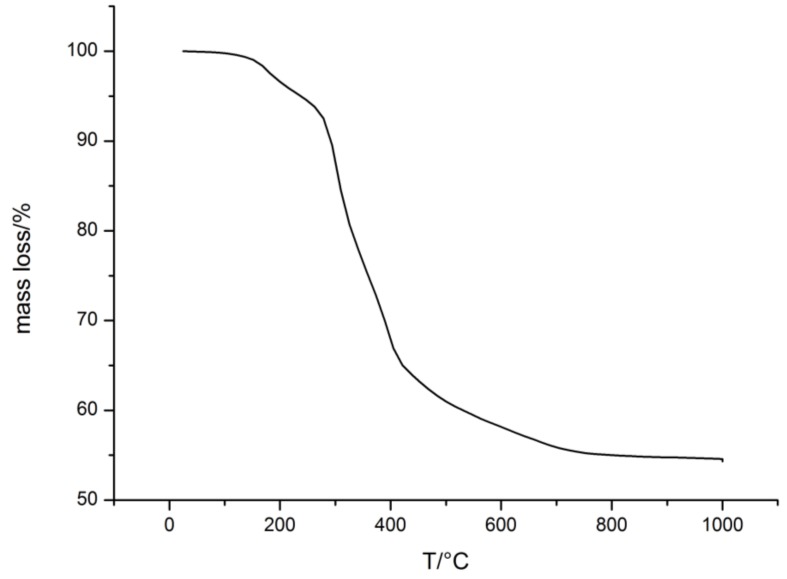
Mass loss of the colloidal crystal made of particles from ATRP during the thermal treatment.

**Figure 12:**
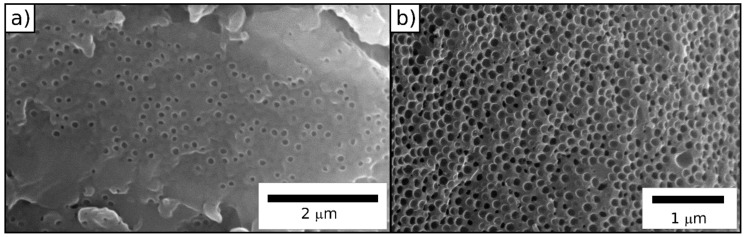
SEM photographs of the (**a**) surface and (**b**) cross-section of a porous SiCN film after melt-shearing and ceramization of core/shell particles processed by emulsion polymerization and ATRP.

TEM image of the core–shell particles obtained after ATRP in THF revealed that the inimer-containing core-particles feature a closed shell surrounding the pristine organic core particles. In other words, an immobilized polysilazane shell was successfully formed. Analogously to the FRP systems in the previous section, hybrid core–shell particles from emulsion polymerization and ATRP were processed into a colloidal crystal stack through melt-shear organization at 160 bar and 160 °C. In a subsequent step, part of the particle-based hybrid film (about 15 mg) was converted into porous ceramics through thermal treatment (Figure 11, TGA measurement of the ceramization process, SEM images in Figure 12).

Referring to TGA measurement, degradation of the polymer core particles starts at ca. 200 °C. This measurement also shows degradation at 500–700 °C that is assumed to result from non-cross-linked silazane oligomers. The ceramic yield amounts to ca. 54 % which shows good conformity to the applied core-ration of 60 wt.%.

SEM images (Figure 12) of the colloidal crystal films after thermal treatment prove the successful preparation of an ordered porous ceramic. In analogy to the ceramics prepared from hybrid core/shell particles obtained after FRP, the surface was partially closed, while the cross-section showed an open-porous structure. The diameter of the pores was determined to be 98 nm, which was in good agreement with expectations taking the material shrinkage during the ceramization process into account.

## 3. Materials and Methods

Methyl methacrylate (MMA) and allyl methacrylate (ALMA) were obtained from Fisher Scientific (Schwerte, Germany), and Dowfax 2A1 from Dow Chemicals (Midland, MI, USA). Durazane1800 was purchased from Merck (Merck, Darmstadt, Germany). All other chemicals were obtained from Sigma-Aldrich (Munich, Germany). For emulsion polymerization of the monomers, the radical inhibitors have to be removed. This was performed by passing the monomers through a basic alumina column. Cu(I)Br was washed five times with glacial acetic acid and ethanol. The solvents used for the free radical polymerization or ATRP were degassed using the freeze–pump–thaw method three times. *N*-,*N*-,*N*′-,*N*′-,*N*″-Pentamethyldiethylenetriamine (PMDETA) was degassed and stored in a glovebox. The copper complexes were freshly prepared in anisole and handled inside the glovebox. All other substances were used as received.

### 3.1. Synthesis of P(MMA-co-ALMA) Particles for FRP

The core particles were synthesized in a 1 L double-wall reactor equipped with a stirrer and a reflux condenser under argon atmosphere. A dispersion of 440 g water, 2.8 g MMA, 1.2 g ALMA, and 50 mg sodium dodecylsulfate (SDS) was filled into the reactor for seed particle synthesis. The emulsion polymerization was initiated by a subsequent addition of 50 mg sodium disulfite, 150 mg sodium persulfate, and 50 mg sodium disulfite, each dissolved in 5 mL water. After 15 min, a monomer emulsion consisting of 19.6 g MMA, 8.4 g ALMA, 36 g water, 90 mg SDS, 70 mg Dowfax 2A1, and 100 mg KOH was continuously added with 1 mL min^−1^ using a rotary piston pump reglo-CPF digital, RH00 (Ismatec, Wertheim, Germany).

### 3.2. Synthesis of P(MMA-co-ALMA)@P(ALMA-co-BBEM) for ATRP

For the inimer-containing particles, P(MMA-*co*-ALMA) cores were synthesized as described above. Ten minutes after adding the first monomer emulsion, a second monomer emulsion was continuously added with 1 mL min^−1^. The emulsion consisted of 7.9 g BBEM, 1.4 g ALMA, 25 g water, 150 mg SDS, and 150 mg Dowfax2A1.

### 3.3. Functionalization with Durazane1800 Using FRP

For the functionalization of the particles with Durzane1800 using FRP, 1.0 g of the lyophilized P(MMA-*co*-ALMA) particles was placed in a Schlenk tube and dissolved in 20 mL toluene. After the addition of 1.3 mL Durazane1800, the reaction mixture was heated up to 100 °C. For the initiation of the polymerization, 0.3 mL di-*tert*-butyl peroxide was added. After 4 h, the reaction mixture was cooled down in an ice bath, and toluene was evaporated under vacuum. The resulting gel was dried under vacuum at 80 °C to receive completely dried particles.

### 3.4. Functionalization with Durazane1800 Using ATRP

For the functionalization of the particles with Durzane1800 using ATRP, 1.0 g of the lyophilized inimer-containing particles were placed in a Schlenk tube and dissolved in 20 mL anisole or THF. After the addition of 1.3–6 mL Durazane1800, the reaction mixture was heated up to 90 °C (anisole) or 55 °C (THF). For the initiation of the polymerization, 0.2 mL [Cu^I^(PMDETA)Br] were added. After 72 h, the reaction mixture was cooled down in an ice bath. In the case of the usage of anisole, particles were precipitated in methanol by using THF, and particles were isolated through evaporating THF under vacuum and the particles were completely dried under vacuum at 80 °C.

### 3.5. Melt-Shear Organization

For the preparation of hybrid opal films, the obtained particle powder were enclosed between two polyethylene terephthalate (PET) foils and inserted into a Collin laboratory press P200 P/M (Dr. Collin GmbH, Ebersberg, Germany). The films were produced by a pressing process at 160 °C and 160 bar for 3 min.

### 3.6. Characterization

Transmission electron microscopy (TEM) measurements were carried out on a Zeiss EM10 (Zeiss, Oberkochen, Germany) electron microscope operating at 60 kV and a Zeiss EM10CR with an Olympus Megaview 2 camera and the iTEM (build) 1276 for camera control software. The images were obtained with a slow-scan CCD camera from TRS (Tröndle, Moorenweis, Germany) in brightfield mode. For dynamic light scattering (DLS) measurements, a Zetasizer ZS90 equipped with a 4 mW 633 nm He–Ne (Malvern Instruments, Malvern, UK) was used. For a typical determination, three measurements with 15 runs were carried out in water as dispersion medium with a solid content of about 0.12 ppm (1.2 × 10^−7^ wt.%). Measurements were performed at 25 °C with an equilibration time of 120 s and a sample volume of about 1–1.5 mL. Automated data acquisition was carried out using a cumulant fit. Scanning electron microscopy (SEM) was performed with a XL30 FEG (FEI/Philips, Hillsboro, OR, USA), with accelerating voltages between 15 and 30 kV. The SEM samples are previously coated with gold for 100 s at 30 mA using a Q300T D (Quorum, Laughton, UK) sputter coater. TGA measurements were conducted with a TGA 2 (Mettler Toledo, Columbus, OH, USA).

## 4. Conclusions

In conclusion, the demonstrated work shows an efficient way to produce SiCN ceramics with ordered macropores. As a precursor for the ceramics, the preceramic polymer Durazane1800 was used while methacrylate-based colloids conduced as pore templates. At first, in order to obtain hybrid organic/inorganic core–shell particles, starved-feed emulsion polymerization was combined with other polymerization techniques, such as free radical polymerization and ATRP. Regarding the melt-shear organization process, it is important to synthesize strongly cross-linked core particles. At the same time, the network based on Durazane1800 is only weakly cross-linked and a too extensive curing of this material was avoided. This leads to a meltable shell, which is also a requirement for melt-shear organization. Melt-shear organization transforms the core–shell particles into free-standing colloidal crystal, while ceramization leads to macroporous SiCN ceramics through degradation of the polymer cores.

Both processes, FRP and ATRP, allow the binding of Durazane1800 on the surface of different monodisperse particles prepared via emulsion polymerization. As it is shown in this work, the synthesized hybrid particles were suitable for melt-shear organization and delivered opal films, which were successfully converted into porous ceramics by thermal removal of the polymer-based core particles.

The present work shows an efficient way to process SiCN ceramics with ordered macropores, which uses a preceramic polymer, i.e., a polysilazane, as precursor for the SiCN ceramic and methacrylate-based colloids conduce as pore templates.

Further work in the field of macroporous SiCN ceramics will be concerning the specific surface areas and mechanical properties, like high temperature or oxidation resistance, of the fabricated materials. In our opinion, as only small amounts of the hybrid material were converted into ceramic material, possible applications for the prepared ceramics lies especially in the field of catalysis. We envisage that thermal treatment of larger particle-based hybrid polymer films can lead to porous ceramics for the preparation of functional porous ceramic membranes for separation processes.

## Figures and Tables

**Table 1:** Average hydrodynamic diameter of particles measured by means of dynamic light scattering (DLS) and average.

Method	Diameter (nm)	*Ð*
DLS	147.8 ± 2.1	0.042
TEM	128.9 ± 1.6	-

Diameters of the dried particles, as determined by TEM. In the case of TEM analysis, 60 particles were measured with respect to their size.

**Table 2:** Average hydrodynamic diameter of particles measured by means of DLS and average.

Method	Diameter (nm)	*Ð*
DLS	201.2 ± 4.4	0.008
TEM	183.7 ± 6.7	-

Diameters of the dried particles, as determined by TEM. In the case of TEM analysis, 60 particles were measured with respect to their size.

## Supplementary Materials

The following are available online at XXX: Figure S1: Photography of a film out of organic particles mixed with Durazane1800 and processed by melt-shear organization; Figure S2: DLS measurement of P(MMA-*co*-ALMA) particles.
