# Supplementary material for: Combining Soft Polysilazanes with Melt-Shear Organization of Core–Shell Particles: On the Road to Polymer-Templated Porous Ceramics
Source: Molecules. 2019 Sep 30;24(19):3553. doi: 10.3390/molecules24193553 (PMC6803923; doi:10.3390/molecules24193553)
Supplement: Supplementary file 1 [file molecules-24-03553-s001.pdf]

Supplementary Materials

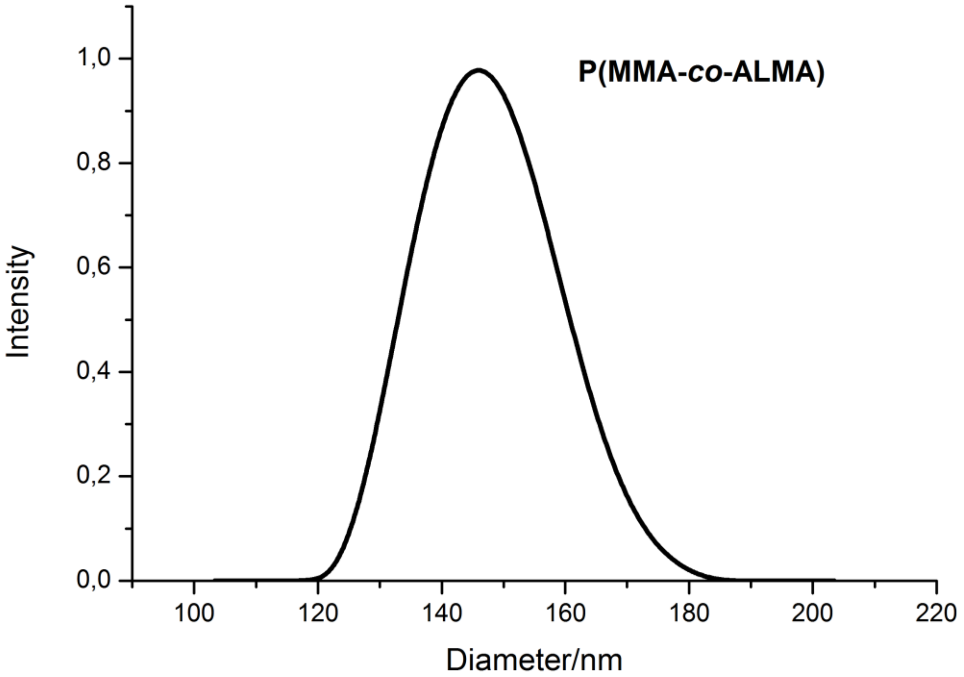

Figure 1. DLS measurement of P(MMA-*co*-ALMA) particles giving a hydrodynamic diameter of 147,8 ± 2,1 nm.

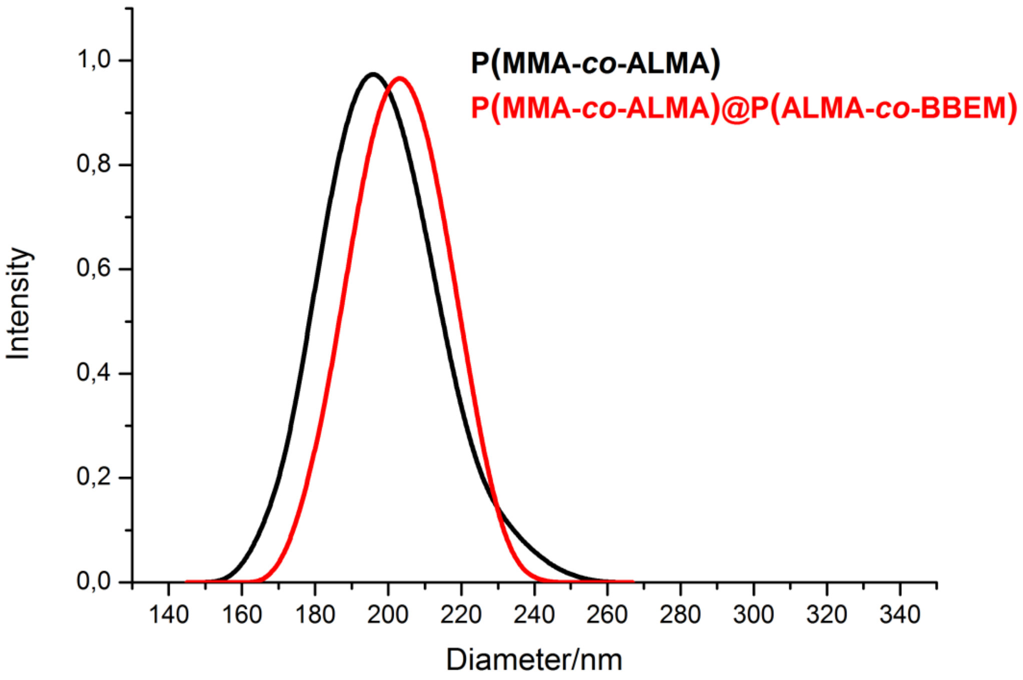

Figure 2. DLS measurement of of inimer-containing particles particles giving a hydrodynamic diameter of 201,2 ± 4,4 nm.
